# In vivo restoration of dystrophin expression in mdx mice using intra-muscular and intra-arterial injections of hydrogel microsphere carriers of exon skipping antisense oligonucleotides

**DOI:** 10.1038/s41419-022-05166-0

**Published:** 2022-09-09

**Authors:** Shani Attias Cohen, Orit Bar-Am, Claudia Fuoco, Galit Saar, Cesare Gargioli, Dror Seliktar

**Affiliations:** 1 Faculty of Biomedical Engineering, Technion–Israel Institute of Technology, Haifa, Israel; 2 Department of Biology, Rome University Tor Vergata, Rome, Italy; 3 Biomedical Core Facility, Faculty of Medicine, Technion-Israel Institute of Technology, Haifa, Israel

**Keywords:** DNA and RNA, Oligo delivery, Preclinical research

## Abstract

Duchenne muscular dystrophy (DMD) is a genetic disease caused by a mutation in the X-linked Dytrophin gene preventing the expression of the functional protein. Exon skipping therapy using antisense oligonucleotides (AONs) is a promising therapeutic strategy for DMD. While benefits of AON therapy have been demonstrated, some challenges remain before this strategy can be applied more comprehensively to DMD patients. These include instability of AONs due to low nuclease resistance and poor tissue uptake. Delivery systems have been examined to improve the availability and stability of oligonucleotide drugs, including polymeric carriers. Previously, we showed the potential of a hydrogel-based polymeric carrier in the form of injectable PEG-fibrinogen (PF) microspheres for delivery of chemically modified 2′-O-methyl phosphorothioate (2OMePs) AONs. The PF microspheres proved to be cytocompatible and provided sustained release of the AONs for several weeks, causing increased cellular uptake in mdx dystrophic mouse cells. Here, we further investigated this delivery strategy by examining in vivo efficacy of this approach. The 2OMePS/PEI polyplexes loaded in PF microspheres were delivered by intramuscular (IM) or intra-femoral (IF) injections. We examined the carrier biodegradation profiles, AON uptake efficiency, dystrophin restoration, and muscle histopathology. Both administration routes enhanced dystrophin restoration and improved the histopathology of the *mdx* mice muscles. The IF administration of the microspheres improved the efficacy of the 2OMePS AONs over the IM administration. This was demonstrated by a higher exon skipping percentage and a smaller percentage of centered nucleus fibers (CNF) found in H&E-stained muscles. The restoration of dystrophin expression found for both IM and IF treatments revealed a reduced dystrophic phenotype of the treated muscles. The study concludes that injectable PF microspheres can be used as a carrier system to improve the overall therapeutic outcomes of exon skipping-based therapy for treating DMD.

## Introduction

Duchenne muscular dystrophy (DMD) is the most common and severe form of muscular dystrophy. It is caused by a gene mutation that inhibits the expression of dystrophin protein [1]. Dystrophin is an essential protein that connects the cytoskeleton to the muscle sarcolemma; any loss in dystrophin expression is characterized by muscle inflammation and degeneration, eventually replacing the muscle tissue with fibrotic tissue and fat [2]. There are no cures for DMD and the current standard of care for the disease is corticosteroids, which result in side effects including weight gain, nervous system disturbances, gastrointestinal symptoms and osteoporosis [3]. There are several new therapeutic strategies on the horizon to cure DMD, including gene therapy [4–6]. One of the most promising gene altering strategies to treat DMD is exon skipping, a mechanism that is regulated by antisense oligonucleotides (AONs) [7]. The AONs are targeted to a specific pre-mRNA region to restore the disrupted reading frame, which eventually produces a shorter transcript of a more functional dystrophin protein [8].

Applying AONs for DMD requires better stability and improved cellular uptake of the oligonucleotides to ensure efficacy in the targeted muscle tissue. The stability of AONs has been markedly enhanced using chemical modifications to the nucleotide backbone. The most prevalent modifications include the 2′-O-methyl–modified oligoribonucleotides with a uniform phosphorothioate backbone (i.e., 2OMePS) [9–11] and phosphorodiamidate morpholino oligomers (i.e., PMO) [12–14]. Modified AONs targeting the dystrophin protein has been widely studied over the past few years, showing encouraging results in both pre-clinical studies using a DMD mouse model (i.e., *mdx* mice) and in human clinical trials [15–17]. One challenge that remains is the low tissue uptake, which impacts the therapeutic benefit of this approach. The current strategy for AON delivery is premised on a systemic administration of the modified AONs, but a couple of recent studies have concluded that these platforms are inefficient in delivering the AONs to an adequate portion of muscle mass for dystrophin restoration [18, 19]. The main problem with systemic administration of AONs for DMD is that it does not selectively target the diseased muscle. Therefore, improved efficacy can be achieved simply by better targeting the AONs using localized gene delivery paradigms.

Hydrogels are a class of biomaterials that have been extensively investigated in the context of gene delivery. A number of studies have reported on the use of biodegradable polymeric hydrogels for the encapsulation of therapeutic RNA or DNA, with release profiles mediated by the carrier degradation [20, 21]. In this context, both natural and synthetic polymers have been used, including hyaluronic acid [22–25], alginate [26], gelatin [27], poly(ethylene glycol) (PEG) [28, 29] and Pluronic [30, 31]. Recent efforts to design hydrogel-based gene delivery systems have focused on enhanced stability, improved sustained release, and more effective pharmacokinetic properties of the therapeutic oligonucleotides. Our laboratory uses a semi-synthetic PEG-fibrinogen (PF) hydrogel for controlled gene delivery [32]. The PF hydrogels are prepared into injectable microspheres that contain 2OMePS modified AONs. The AONs are complexed with polyethyleneimine (PEI), which serves as a transfection agent with the purpose of increasing the pharmacokinetic properties and stability of the AONs [33–35]. The PF hydrogel microspheres were fabricated by a dual photo-initiator technique which produces microspheres with entrapped AONs having a characteristic size that is sufficiently small for injectability through a 25-gauge syringe [36, 37]. We previously showed that these injectable AON-laden PF microspheres exhibited a sustained release profile of the entrapped 2OMePS AONs, mediated by the biodegradation property of the hydrogel [32]. We documented that AONs released from the biodegrading PF microspheres were well-penetrated in myoblasts isolated from *mdx* mice. In a preliminary in vivo study, muscles of *mdx* mice were injected intramuscularly (IM) with the biodegradable AON-laden PF microspheres. The local in vivo biodegradation of the PF implants was documented using a Gadolinium-labeling technique combined with Magnetic Resonance Imaging (MRI). The results indicated resorption of the microspheres within 4 weeks after implantation, which coincided with a significant therapeutic benefit as measured by a reduction in inflammation when compared to control groups.

In this study, we aim to further investigate the efficacy of the PF delivery system using an intra-arterial approach for treating DMD. Gene delivery through local IM injections can be challenging in whole-limb correction, such as required in dystrophic patients [38, 39]. Recent studies on IM injected therapeutic AONs showed the limitations of this administration route, namely multiple repeat injections of the AONs to establish dystrophin restoration [40–42]. We speculate that an intra-arterial delivery approach may provide better efficacy from AON-laden controlled-release microspheres that target muscle tissue, without the need for multiple injections. Previous studies have shown the distinct benefits of using intra-arterial injection to treat dystrophic mice using delivery of either mesoangioblasts [43], or modified tendon fibroblasts expressing an angiogenic factor [44]. Here we sought to apply the intra-arterial administration to deliver the 2OMePS because this would allow enhanced transduction of the whole limb. Accordingly, we speculate that intra-arterial injections of AON-laden PF microspheres will increase the effectiveness of the treatment as compared to IM delivery.

Hence, we set out to perform an in vivo study comparing intra-arterial versus IM efficacy of AON therapy in DMD. Delivery of 2OMePS/PEI polyplexes loaded in PF microspheres was assigned to either an IM or intra-femoral (IF) injection group of *mdx* mice. We hypothesized that injection of the microspheres into the femoral artery would result in more uniform transduction of the limbs downstream of the injection site, which will in turn result in improved efficacy. Moreover, IF administration should enable the injected microspheres to reach a higher muscle area as compared to the more localized IM injection, thereby reducing the needed number of injections to treat each muscle group.

In the present study, we examined the restoration of the expression of dystrophin in *mdx* mice after IM and IF injections of 2OMePS loaded PF microspheres. We also documented localization and biodegradation profiles of the microspheres up to 28 days post-injection in the muscles using MRI. Quantitative polymerase chain reaction (qPCR) was used to demonstrate higher exon skipping after IF injection, supporting our hypothesis that intra-arterial administration enhances the efficacy of the AONs. Importantly, this study provides strong evidence that injectable PF microspheres can be an effective vehicle for AON delivery, with potential use for exon skipping-based therapy for DMD.

## Materials and methods

### 2OMePS polyethyleneimine polyplexes

Complexes (polyplexes) were formed according to a procedure described by Boussif et al. [45]. Briefly, 2OMePS- Polyethyleneimine (PEI) polyplexes were prepared at different N/P ratios (ratio of the number of nitrogen groups on the polymer to the number of phosphate groups on the backbone of the AONs). The N/P ratios were calculated based on PEI nitrogen per nucleic acid phosphate (1 µg of DNA/RNA is equivalent to 3 nmol of phosphate). To form polyplexes, different amounts of 2OMePS were diluted in 200 µL of 150 mM NaCl solution (Sigma Aldrich). The desired amount of Polyethylenimine (PEI; Mw 25 kDa, Mn 10 kDa, branched, Sigma Aldrich) was taken from 150 mM PEI solution, at an N/P ratio of 10 and 15, and diluted into a separate solution of 150 mM NaCl (200 µL). The PEI solution was added to the 2OMePS solution and vortexed for 15 s. The resulting polyplexes were incubated for 30 min at room temperature before use.

### PEG-fibrinogen microsphere synthesis

**Fig. 1:**
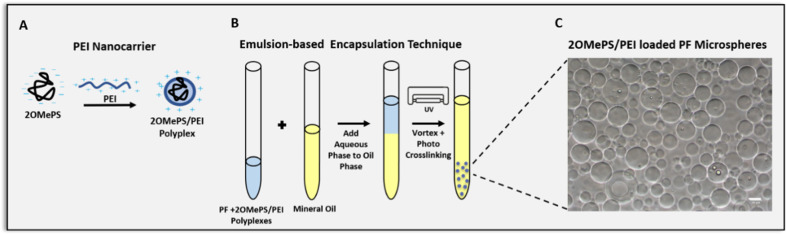
PF microspheres made with an emulsion-based technique for 2OMePS AON encapsulation. **A** The 2OMePS AONs were complexed with a transfection carrier, PEI, to form nano-scaled polyplexes. **B** The resultant polyplexes were encapsulated using a dual photo-initiator emulsion-based technique to form AON-laden PEG-fibrinogen (PF) microspheres**. C** A representative light microscopy image of PF microspheres, (scale bar = 50 µm).

PEG-fibrinogen (PF) was prepared by a Michael-type addition reaction of PEG-diacrylate (PEG-DA, 10-kDa, Fluka, Buchs, Switzerland) with bovine fibrinogen (ID bio, France), as previously described [46]. Microspheres of PF were formed using a dual photoinitiator water-in-oil emulsion method based on previously reported procedures described by Franco et al*.* [37]. Briefly, 20 µg 2OMePS antisense oligonucleotides (AONs) (5′-UCCAUUCGGCUCCAAACCGG3′, IDT) were used to create PEI polyplexes; the polyplexes were dissolved in phosphate-buffered saline (PBS) and added to 1 ml of PF hydrogel precursor solution containing 2% additional PEG-DA (10-kDa, w/v), 0.15% (v/v) triethanolamine (TEOA), 2.5% (v/v) Irgacure®2959 photoinitiator stock solution made from 100 mg/ml in 70% ethanol, and 1% Pluronic F-127 (w/v) solution in PBS. The aqueous phase PF solution (200 µl) was added to 1 ml of oil phase solution consisting of mineral oil (Sigma Aldrich), 0.5% (v/v) Irgacure®651 photoinitiator, and 1.5% (v/v) TEOA in a glass test tube. The two phases were vortexed using a Vortex-Genie 2 (Scientific Industries Inc., Bohemia, New York, USA) at full speed for four seconds, and immediately exposed to UV-light (365 nm, 5 mW/cm^2^) for another 26 s for photo-crosslinking to create the PF hydrogel microspheres (Fig. 1). The emulsion was washed and resuspended three times in 1 ml PBS (pH 7.4) and centrifuged at 1200 g for 4 min to separate the aqueous and oil phases. The oil phase was aspirated, and the remaining aqueous phase was resuspended in PBS.

### Cell culture

Extraction and use of animal tissue were approved by the Israel Animal Board and Safety Committee (approval number IL-18-02-32). Primary mouse myogenic progenitors from *mdx* mice were extracted from eight week old mice as was previously described [47, 48]. The hind-limb muscles of *mdx* mice, which were kindly donated from A.D.I Association Duchenne, Israel, were isolated, and the fat and bones were discarded. The muscles were minced with scissors, enzymatically dissociated at 37 °C with 0.05% trypsin-EDTA for 30 min, and centrifuged at 2500 rpm for 5 min. The cells were collected, and the trypsinization of the remaining undigested tissue was repeated twice by adding fresh trypsin solution. On the fourth repeat, the cells were incubated for 30 min with 0.25% trypsin-EDTA. After centrifugation, the cells were suspended in a proliferation medium, BIO-AMF-2 (Biological Industries Ltd.), on Matrigel-coated plates (Cultrex RGF Basement Membrane Extract, R&D Systems, Inc., Minneapolis, MN, USA).

### Delivery of 2OMePS loaded PF microspheres to mdx mouse myotubes

Myotubes from myogenic progenitors taken from *mdx* mice were cultured in BIO-AMF-2 medium and plated at a cell density of 50,000 cells per well in a 24-well plate coated with Cultrex RGF Basement Membrane Extract (R&D Systems, Inc.) at 37 °C under 5% CO_2_. For myotube formation, the media was replaced after 24 h with a differentiation medium (DMEM + 5% horse serum) for 3 days. The resultant myotubes were treated with PF microspheres loaded with 2 µg of 2OMePS/PEI polyplexes per well or with free 2OMePS/PEI polyplexes (1 µg/well) and incubated for 72 h. Control groups of cells treated with empty PF microspheres and untreated myotubes were stained for comparison (*n* = 3).

### In vitro dystrophin immunostaining

The transfected *mdx* myotubes were examined for dystrophin expression with a polyclonal rabbit antibody against the dystrophin carboxyl-terminal region (1:100; ab15277, Abcam, UK). Polyclonal antibodies were detected by donkey-anti-rabbit immunoglobin G Alexa Fluro 647 (1:400; Molecular probe, Invitrogen, UK) and nuclei were detected using DAPI staining (1:1000, Sigma Aldrich). Control WT C-57BL6 mouse myotubes were kindly provided from the laboratory of Professor Shulamit Levenberg, Technion, and stained similarly for dystrophin expression. The stained myotubes were imaged using a Zeiss LSM 700 confocal microscope (Carl Zeiss, Oberkochen, Germany). All images were sampled at a resolution of 1024 by 1024 pixels using a 20× and a 40× objective, and scanning areas were randomized.

### Animals studies

All mice experimental procedures were performed according to the good animal experimentation guidelines I.A.C.U.C. No. 432 of March 12–2006 and upon authorization from the Italian Ministry of Health, protocol #20/01-D.

### In vivo injections of PF microspheres

IM injections were performed on *mdx* dystrophic mice (6–8 weeks of age). The tibialis anterior (TA) and Gastrocnemius (GA) muscles of the animal (bilateral; *n* = 4) were injected with 20 µl or 30 µl of PF microspheres loaded with 2OMePS/PEI polyplexes, respectively. Control groups included empty PF microspheres (*n* = 3) and saline containing 1 µg of 2OMePS/PEI free polyplexes (*n* = 3) that were injected directly into the muscles with the same volume as the treatment group. The administration of all treatments was done through the skin into the proximal and mid-belly portions of the muscles using a 30-gauge syringe needle. At 30 days post-injection, the TA and GA muscles were isolated, snap-frozen in liquid nitrogen, and stored at −80 °C. Additional muscles were harvested from un-injected age-matched *mdx* control and normal C-57 mice.

For IF injection, the femoral artery was injected as described previously [44]. Briefly, a limited incision on the medial side of the leg was performed and the femoral artery was gently separated from surrounding muscle and connective tissue. The needle of an insulin syringe was inserted into the femoral artery and PF microspheres loaded with 2OMePS/PEI polyplexes (*n* = 6) were delivered slowly (approximately 30–40 s) in a volume of 30–40 µl. There was no visible damage to the vessel wall during or after the operation. The mice were sacrificed at 20 days and 40 days post-injection. The TA and GA muscles were isolated and snap-frozen in liquid nitrogen, and immediately stored at −80 °C. Non-treated mice muscles were isolated for control at the same time points (*n* = 6). Data analysis were done in a non-blinded fashion.

### In vivo imaging

PF precursor solution was pre-labeled with gadolinium (Gd) as described elsewhere [49] to provide contrast for magnetic resonance imaging (MRI). For both in-vitro and in-vivo imaging, MRI was performed on a 9.4 T scanner (Bruker Biospec, Ettlingen, Germany), using a transmit/receive cylindrical volume coil (86 mm diameter). For calibration of the MRI signal in vitro, different dilutions (20–100%) of the PF-Gd microspheres were prepared in PBS solution and T1 maps were acquired using a Rapid Acquisition with Relaxation Enhancement (RARE) pulse sequence with variable repetition time. The parameters used were as follows: TR = 100, 200, 300, 500, 800, 1000, 2000, 3000, 4000, 5000, 6000, 7000, 8000, 9000, 10,000 ms, TE = 25 ms, RARE factor = 8, 2 mm slice thickness, 250 µm in-plane resolution, field of view (FOV) = 4.0 × 4.0 cm^2^, matrix size = 160 × 160, scan time = 15 min. T1-map images were calculated by performing exponential curve fitting for each pixel using a custom build software in Matlab (MathWorks, MA). R1 values were calculated from T1 values by R1 = 1/T1 (Fig. S2). For in vivo MRI, animals were anesthetized with 0.5–1.5% isoflurane supplemented with oxygen (0.6 l/min). Respiration was monitored during imaging using a respiration monitor (Small Animal Instruments, Stony Brook, New York, NY) and body temperature was maintained using circulating hot water. PF-Gd microspheres (30 µl, 50% v/v) were injected into the GA, TA, and quadriceps muscles of the WT C-57 mouse (*n* = 6). MRI was performed at different time points using a 9.4 T Biospec spectrometer with a quadrature RF volume coil. T1-weighted images of the mouse lower body were acquired using a fast-low angle shot (FLASH) sequence with respiratory gating, and 150 µm in-plane resolution, 0.35 mm slice, TR/TE = 5.8/2.7 ms, 10° pulse, FOV = 6 × 4 cm^2^, matrix size = 400 × 266, and 15 repetitions; Total scan time ~10 min. Data processing was done using Medical Image Processing, Analysis, and Visualization (MIPAV) software (NIH; http://mipav.cit.nih.gov). The Gd-bead volume for the muscles in each mouse was measured by manually segmenting each area and calculating the volume over time. ROIs were later drawn, the intensity was measured over time and the signal intensity was normalized to the noise (Figs. S2 and S3).

### Exon skipping evaluation

Total RNA was isolated from snap-frozen GA muscles using TRIzol reagent according to the manufacturer’s instructions (Invitrogen, UK). Nested RT-PCR was carried out with 200 ng of total RNA as the starting material for 25 cycles of amplification in a One-step RT-PCR kit (QIAGEN, Germany). Briefly, forward and reverse primers amplifying from exons 20 and 26, respectively (Ex20Fo, 5′-CAGAATTCTGCCAATTGCTGAG-3′; Ex26Ro, 5′-TTCTTCAGCTTGTGTCATC-C-3′) were used at an annealing temperature of 55 °C for 1 min with an extension time of 2 min at 72 °C. The RT-PCR product (2 µl) was used as a template in a 50 µl secondary nested PCR with DreamTaq^TM^ Hot Star Green PCR master mix (ThermoFisher scientific) and inner primers (Ex20Fi, 5′-CCCAGTCTACCACCCTATCAGAGC-3′; Ex26Ri, 5′-CCTGCCTTTAAGGCTTCCTT-3′). The secondary PCR was carried out for 25 cycles. The cycle conditions were 95 °C for 30 s, 57 °C for 30 s, and 72 °C for 2 min. PCR products were then examined by electrophoresis on a 2% agarose gel.

For qPCR, 5 µg RNA was used for cDNA synthesis using a High-Capacity cDNA Reverse Transcription Kit (Applied Biosystems, CA) according to the manufacture’s protocol. Levels of DMD exon 23 skipping were determined by multiplex qPCR of HEX-labelled primers spanning Exon 6–7 (Assay Mm.PT.58.43996802, Integrated DNA Technologies (IDT), Belgium) and FAM-labelled primers spanning Exon 23–24 (Mm.PT.47.7668824, IDT, Belgium). The results were normalized to mouse Tbp housekeeping gene (Mm.PT.39a.22214839, IDT, Belgium). The cDNA (60 nanograms) was used as an input per reaction and all assays were carried out in triplex with PrimeTime^®^ Gene Expression Master Mix (1055772, IDT, Belgium). Assays were performed under fast cycling conditions on the QuantStudio1 (Applied Biosystems, Thermo Fisher Scientific) and all data were analyzed using the comparative Ct method. For a given sample, the delta-delta-Ct values of exon 6–7 and exon 23–24 assays were used to calculate a relative abundance of total dystrophin and exon 23-skipped dystrophin mRNA, respectively. Exon 23 skipping was then expressed as a percentage against total dystrophin, as indicated by the exon 6–7 expression level.

### Protein extraction and western blotting

Tissue samples of GA and Quad were homogenized in ice, mixed with lysis buffer (50 mmol/l Tris/HCl, pH 7.4, 1 mmol/l EDTA, 1 mmol/l EGTA, 1% Triton X-100, 1 mmol/l), and protease inhibitor cocktail (Sigma-Aldrich), and separated by centrifugation at 11,000 g for 20 min at 4 °C. Protein concentrations were determined by bradford protein assay (Pierce Biotechnology Inc., Rockford, IL, USA) using BSA as a standard. For western blotting analysis, 50 µg of protein were transferred to membranes (Immobilon; Amersham Biosciences Inc., Piscataway, NJ, USA), saturated with blocking solution (1% BSA and 0.1% Tween-20 (Sigma-Aldrich) in PBS) and hybridized with cleaved caspase-3 rabbit monoclonal antibody (#9669; Cell Signaling Technology, Danvers, MA, USA), α-SG mouse monoclonal antibody (Ad1/ 20A6; Vector Laboratories) or lacZ polyclonal antibody (#55976; Cappel Laboratories) at 1:1,000 dilution, or with GAPDH monoclonal antibody (GAPDH-71.1; Sigma-Aldrich) at 1:10,000 dilution for 1 h at room temperature. The blots were washed three times (15 min each at room temperature) with blocking solution, and then reacted with anti-mouse or anti-rabbit secondary antibody conjugated with HRP (Bio- Rad Laboratories, Inc., Hercules, CA, USA) at 1:3,000 dilution for 1 h at room temperature. The blots were then washed three times, and finally visualized with an enhanced chemiluminescent immunoblotting detection system (Pierce Biotechnology Inc).

### Immunohistochemistry

Histological sections (8 μm in thickness) were cut from at least two-thirds of all muscles at 100 μm intervals. The sections were then examined for dystrophin expression with a polyclonal rabbit antibody against the dystrophin carboxyl-terminal region (1:100, ab15277, Abcam, UK) and with a monoclonal mouse antibody against dystrophin rod domain (1:20, NCL-DYS1, Leica Biosystems). Polyclonal antibodies were detected by donkey-anti-rabbit immunoglobin G Alexa Fluor® 647 (1:400, Molecular probe, Invitrogen, UK) and Alexa Fluor® 647 Donkey Anti-Mouse IgG (1:100, Jackson Immune Research laboratories), respectively. Laminin was additionally detected using polyclonal rabbit anti-laminin antibody (1:80, L9393, Sigma Aldrich). The maximum number of dystrophin-positive fibers in one section was counted using IMARIS image analysis software and were defined as dystrophin-positive fibers when more than two-thirds of the single fiber showed continuous staining above the NT muscle background threshold value.

### Histology

Histological sections (8 µm in thickness) were cut from at least two-thirds of the TA muscles at 100 µm intervals. Hematoxylin and eosin (H&E) staining was performed according to manufacture protocol (Sigma Aldrich). Images of the stained slides were obtained using 3DHistech Pannoramic 250 Flash III scanner at ×20 magnification (3DHISTECH Ltd., Budapest, Hungary). Select regions within each slide were imaged using an Olympus microscope (BX60, serial NO. 7D04032) fitted with a digital microscope camera (Olympus DP73, serial NO. OH05504) and objectives with magnifications of ×10 and ×20. A semi-quantitative analysis was performed to evaluate the cellular inflammation of the tissue specimen based on a scoring system detailed in the supplementary data. A blinded score for each specimen was based on four possible grades for each parameter (i.e., Grade 0–4, see supplementary), as determined by an independent pathologist. The scoring was calculated by averaging a least 3 slides for each treated muscle, each section was averaged with 3–4 regions of interest (ROIs) where each treatment was represented by at least 3 mice. The percentage of centrally nucleated fibers was determined by analyzing the images and counting the number of centrally located nuclei; a total of at least 100 cells per field of view were evaluated in each image (*n* = 3).

### Statistical analysis

Statistical analysis was performed using the GraphPad Prism 9 software. Data from independent experiments were quantified and analyzed for each variable. All the results were expressed as mean ± standard deviation (SD). Interactions between the groups were tested by two-way ANOVA. In all experiments, the differences were considered statistically significant when *p* < 0.05. The extent of significance was reported as follows: ****p < 0.0001, ***p < 0.001, **p < 0.01, *p < 0.05.

## Results and discussion

PF microspheres were formulated using an emulsion-based dual photo-initiator technique that was modified from Pradhan et al. [36, 50] to deliver 2OMePS AONs in a controlled release manner. The 2OMePS AONs were complexed with PEI, a well-known transfection agent [33, 51, 52], to form nanoscale polyplexes (Fig. 1A) that were added to the PF hydrogel precursor (Fig. 1B). This delivery system was previously characterized by our group [32]. We demonstrated that this technique produced spherical microspheres with an average diameter of approximately 80 μm. Under laboratory conditions, the in vitro sustained release profile of the encapsulated AONs was shown to be affected by the PF hydrogel formulation and enzymatic biodegradation of the microspheres [46, 53]. Furthermore, the 2OMePS released from the microspheres were well-penetrated in *mdx* derived myotubes.

### In vitro dystrophin restoration in *mdx* derived myotubes

**Fig. 2:**
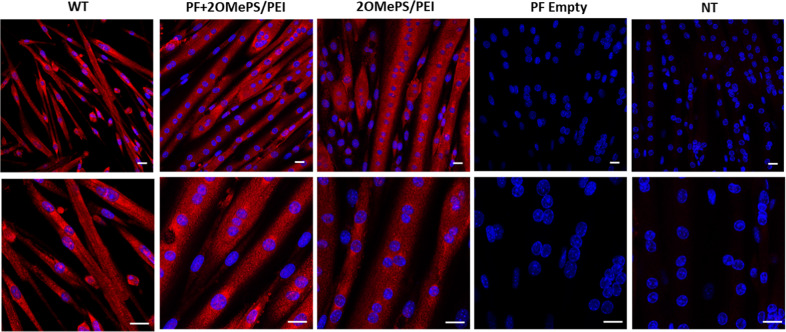
In Vitro dystrophin restoration using AON-laden PF microspheres. Dystrophin immunostaining (red) and DAPI staining (blue) of mdx myotubes after 72 h incubation with PF microspheres loaded with 2 µg of 2OMePS/PEI AON polyplexes. Control groups of free 2OMePS/PEI AON polyplexes were stained and compared to negative controls of non-treated mdx myotubes and mdx myotubes treated with empty PF microspheres. Wild type C-57 mouse myotubes were stained as a positive control, (*n* = 3). Scale bar = 20 µm.

To further investigate the efficacy of the released 2OMePS AONs in vitro, we performed dystrophin immunostaining on *mdx* myotubes treated with 2OMePS loaded PF microspheres (Fig. 2). The *mdx* mouse satellite cells were seeded in a tissue culture plate and left to differentiate into myotubes by replacing the media with a differentiation medium for 3 days. Subsequently, PF microspheres loaded with 2OMePS/PEI polyplexes were incubated with the differentiated myotubes for another 72 h. A strong dystrophin signal was observed after the treatment with PF microspheres, as can be seen in Fig. 2. Negative controls of empty PF microspheres and non-treated myotubes did not show any dystrophin expression—which is expected because the *mdx* mouse does not express dystrophin due to their exon 23 mutation [54]. The *mdx* derived myotubes were also treated with free 2OMePS/PEI polyplexes, as a positive control, which also induced strong dystrophin expression like that observed in myotubes treated with the PF-AON microspheres. The resemblance in the expression of the positive control and the PF-AON treatment indicates that the emulsion-based encapsulation technique did not cause any change that alters the integrity and functionality of the encapsulated 2OMePS/PEI polyplexes. Wild type myotubes were also stained to show normal dystrophin expression by immunofluorescence, which is very similar to the PF-AON treated specimen staining. This outcome demonstrates that the 2OMePS released from the microspheres were able to restore in vitro dystrophin expression in the *mdx* derived myotubes.

### In vivo localization and biodegradation of PF microspheres

**Fig. 3:**
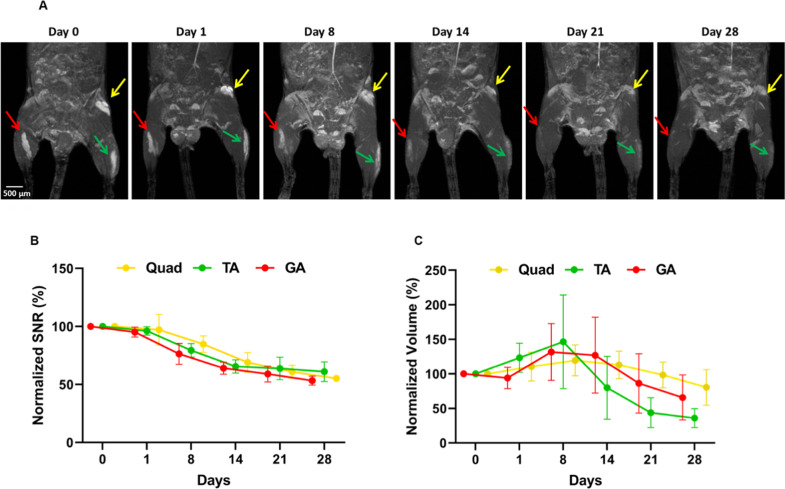
In vivo biodegradation of PF microspheres. The TA, GA, and quadricep muscles were injected with 30 µl each of Gd-PF microspheres and imaged using MRI for up to 28 days. **A** 3D MRI reconstructions of C57-WT mice injected with Gd labeled PF microspheres. Arrows show the location of the implant injected in each muscle group - GA in red, TA in green, and Quadriceps in yellow. **B** Graphical representation of the segmentation data reveals a reduction in the transient Signal to Noise (SNR) parameter. **C** The normalized implant volume parameter exhibits an initial increase followed by a decrease.

The localization and biodegradation of the PF microspheres were evaluated in vivo using MRI. C57BL/6 mice (wt) were injected intramuscularly with PF microspheres labeled with Gadolinium (Gd), which was used as an MRI contrast agent [55]. This in vivo biodegradation assessment for the PF hydrogels was based on a technique first reported by Berdichevski et al. [49]. Consequently, an in vitro calibration of the Gd labeled microspheres with different concentrations in PBS solution was performed prior to the in vivo experiments (Fig. S1). Figure 3 shows 3D reconstruction MR images of WT mice, showing the GA, TA, and quadricep muscles after injection of 30 µl Gd-PF microspheres. The microspheres were detected at several incremental time points post-injection, up to 4 weeks from the implantation. Initially, the microspheres were localized at the target muscles next to the injection site, as shown in Fig. 3A. A reduction of the PF-Gd signal was observed overtime after the injection, irrespective of the muscle group. The gradual reduction in MR signal (SNR) intensity demonstrates a progressive in vivo biodegradation of the PF hydrogel in the muscle tissue. The normalized implant volume was also documented, and initially appears to increase followed by a gradual decrease after the first week post-implantation. The images in Fig. 3A show the maximum intensity projection, MIP, of the implant in each muscle and all the images are shown on the same intensity scale.

The quantification of MRI in Fig. 3B shows the values of SNR and volume based on calculation from acquired 2D images slices from each animal. Each of these slices were manually segmented for each treated muscle (i.e., the MPs in the different muscle slices were identified, segmented manually, and evaluated in terms of calculating the total volume and the intensity for all the selected slices in each muscle). The resultant 3D signal intensity at each time point was normalized with the mean noise signal (see supplementary data). The quantification used is based on methodology that was previously validated. Unlike the SNR parameter, which represents the implanted PF concentration within the tissue, the implant volume is associated with the localization or dispersion of the PF microspheres as they reside in the tissue. Accordingly, the initial increase in volume may be attributed to microspheres that have undergone swelling after their in vivo placement, or microspheres that have slightly dispersed from their injection location. The TA muscle appears to have larger fluctuation in implant volume, when compared to the other muscles. The volume may be affected by the type and location of the muscle in the leg, where movement can influence the dispersion of the microspheres. Taken together, these data indicate that the microspheres are degraded but are still present in the muscle after 4 weeks. Accordingly, residual microspheres would purportedly retain polyplexes and sustained their release to the muscle group during this time.

Although the MRI data confirmed the presence and persistence of the AON-bearing implants in the mouse muscle after injection, these experiments were performed on WT C57BL/6 mice. Dystrophic muscles are physiologically very different from normal muscles in the WT mice. They are characterized by invasion of immune cells and cyclic regeneration/degeneration which may influence the biodegradation of MPs. Future studies should fully explore the biodegradation of the MPs in dystrophic muscles by applying the MRI technique in an *mdx* mouse model. These experiments should evaluate biodegradation at longer longer time-points and include *mdx* and WT mice for side-by-side comparisons, as well as both IM and IF approaches, to provide a more complete picture of the MPs localization and degradation process after injection.

### In vivo administration of 2OMePS loaded PF microspheres

The *mdx* mouse DMD disease model was used in this study to investigate the efficacy of the 2OMePS loaded PF microspheres in vivo. Two routes of administration were used: an IM injection to the Tibialis Anterior (TA) and the Gastrocnemius (GA) muscles and an IF injection (injection into the femoral artery). Previous studies that apply AONs for DMD (without MPs) do so using a minimally invasive systemic approach by intravenous administration, namely by injecting the AONs into the venous circulation [8, 56–58]. The AONs are sufficiently small to be transported through the arterial circulation directly into all the muscles in the body. The use of controlled release MPs limits our ability to use intravenous administration because MPs are large and would be trapped by the lung microvasculature. In order to achieve widespread distribution of MPs in muscle groups, as is characteristic with the systemic administration of AONs, we applied an IF approach. Unlike intravenous administration of AONs, however, the IF approach is a more invasive technique that poses additional risk to the patient. We also examined the IM injection, which is less invasive when compared to IF administration, but results in highly localized implants at the injection site and requires numerous injections for placing MPs into large muscle groups. In principle, the IF injection would distribute the microspheres uniformly throughout the muscle groups downstream of the femoral artery and thus represents the most ideal form of administration using the controlled release MPs. As such, the IF administration route was used for validating the MP technique relative to systemic administration by intravenous injections. Comparisons between IF and IM efficacy, which were evaluated using immunohistochemistry for dystrophin expression and histopathology, helped us to identify advantages and disadvantages of the two approaches in terms of localized versus non-localized effects within the treated muscle.

### Nested PCR and qPCR for exon skipping evaluation

**Fig. 4:**
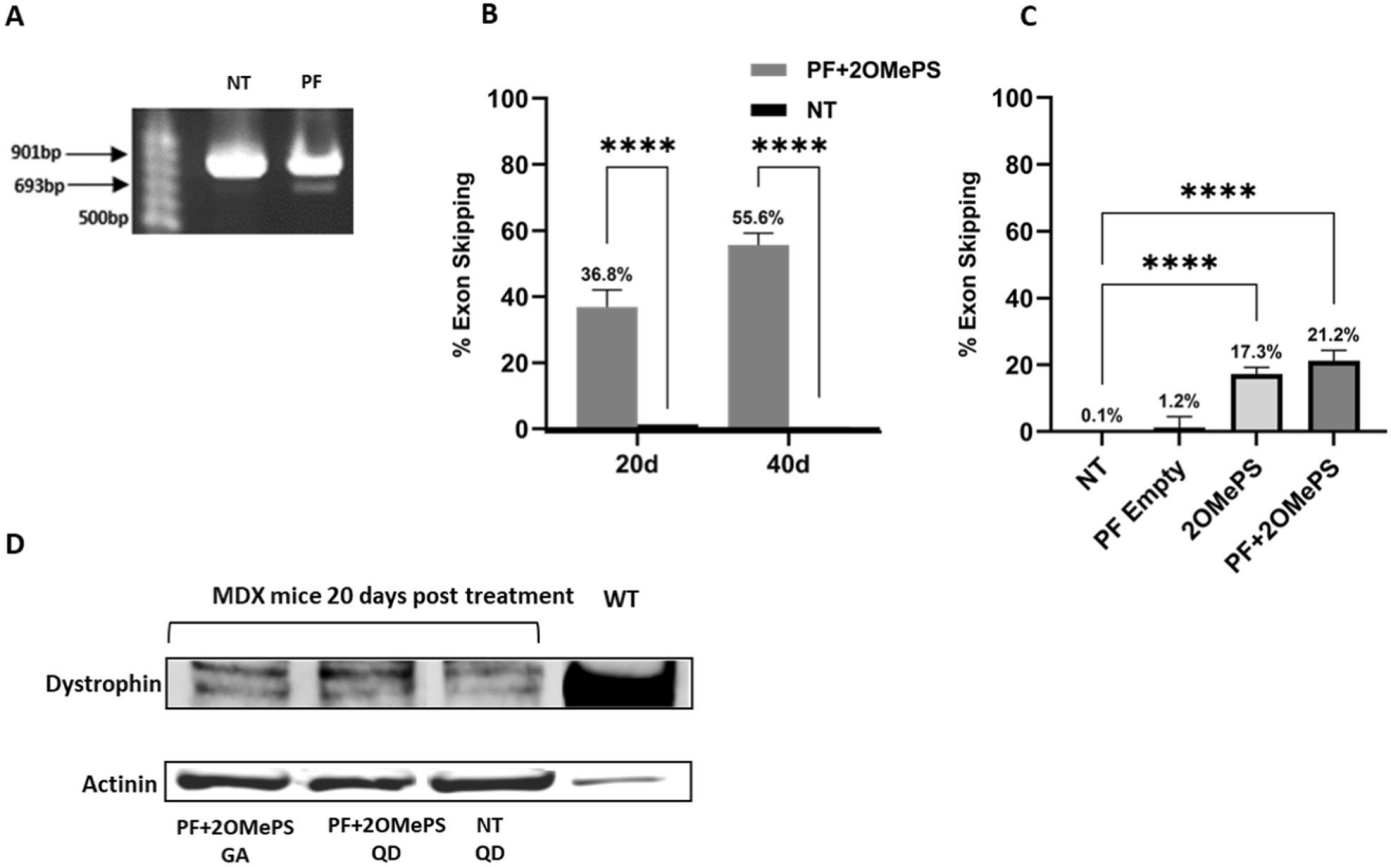
Exon skipping and dystrophin expression. **A** Representative nested PCR of RNA extracted from GA muscle injected intrafemorally (IF) with 1 µg of 2OMePS/PEI polyplexes loaded in PF microspheres. A 901 bp band is observed in the treated group and the non-treated muscle; this band represents the non-skipped product of the PCR. A 693 bp band was observed only in treated muscle; this band represents the truncated product with exon 23 skipped. **B** The PCR products were quantified for the percentage of exon skipping after 20 days and 40 days post-IF injection. **C** Percentage of exon skipping after 30 days post-IM injection. Each PCR reaction was performed in triplicate, ****P < 0.0001 analyzed using an ordinary one-way ANOVA test compared to NT mice. **D** Dystrophin expression of GA and QD muscles measured by WB analysis of mice 20 days post IF injection of 2OMePS loaded PF MPs compared to NT mice and to WT mice. Actinin was used for normalization.

For the detection of exon 23 skipping facilitated by the 2OMePS AONs, nested PCR was performed with primers flanking exon 20 and 26 on the RNA extracted from the GA muscle treated with PF microspheres loaded with 2OMePS\PEI polyplexes (Fig. 4A). A 901 bp band was observed in the treated group and the non-treated muscle; this band represents the non-skipped product of the PCR reaction. A 693 bp band was observed only in treated muscles that underwent exon skipping. This truncated band represents the dystrophin transcripts lacking exon 23. These results are consistent with published data before and after administration of free AONs to treat DMD [8, 56, 57]. The percentage of exon skipping was quantified using RT-qPCR performed using custom-made assays designed against dystrophin exon 6–7, which represents the normal level of dystrophin expression, and the exon 23–24 template, which represents the skipped dystrophin transcript. The percentage of exon 23 skipping was expressed as a percentage of total dystrophin, as indicated by the exon 6–7 expression level after normalization with an endogenous control. Exon skipping of 36.8% was found 20 days post-IF injection and was increased to 55.6% after 40 days. For the IM administration, 30 days post-injection resulted in exon skipping of 21.2%—a truncation efficiency that was significantly lower than that found for 20 days post-IF injection (*p* < 0.01).

This finding reinforces the hypothesis that IF injection enhances the overall efficacy of the 2OMePS AONs treatment using the PF microspheres. Consequently, IF administration of 2OMePS polyplexes was not part of the experimental design because the polyplexes would not selectively reach the targeted muscle group owing to their sub-micrometer size [59–61]. Muscles treated with IM injections of free 2OMePS polyplexes resulted in exon skipping of 17.3%, slightly lower than the exon skipping value found for 2OMePS loaded PF microsphere treatment. This improvement in efficacy for the PF treatment may result from an increased AON stability associated with the encapsulation of the 2OMePS/PEI polyplexes. Additionally, the controlled release of AONs from the microspheres over time may improve their uptake in the muscle tissue, leading to higher reading-frame recovery.

### Dystrophin immunoassaying after IM and IF injections of PF microspheres

**Fig. 5:**
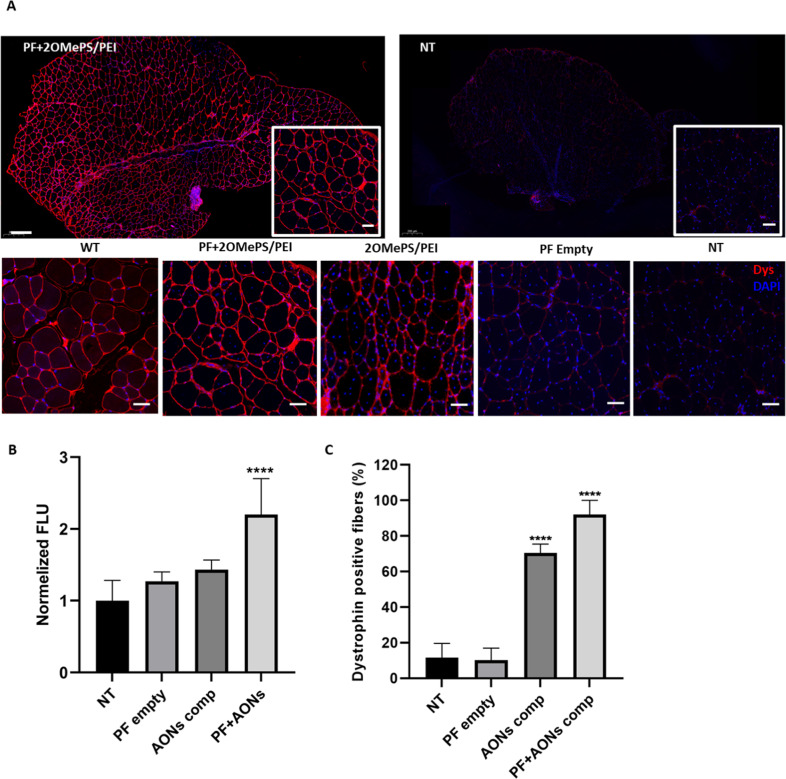
Dystrophin expression after intramuscular (IM) injection. **A** Immunohistochemical staining of dystrophin (red) and DAPI (blue) in the TA muscle of C57 WT mice (WT), treated mdx mice 30 days post intramuscular injection of 2OMePS/PEI loaded PF microspheres (PF + 2OMePS/PEI, *n* = 10), free 2OMePs/PEI polyplexes (2OMePS/PEI, *n* = 7) or empty PF microspheres (PF empty, *n* = 3), and non-treated mdx mice (NT, *n* = 3). Representative images are shown; scale bar = 50 μm. **B** Fluorescence intensity to normalized non-treated mdx mice (NT, *n* = 7) of TA cryosections stained for dystrophin. **C** Percentage of dystrophin-positive fibers after intramuscular (IM) injection of 2OMePS/PEI loaded PF microspheres (PF + AONs comp, *n* = 6), free 2OMePs/PEI polyplexes (AON comp, *n* = 6), or empty PF microspheres (PF empty, *n* = 4) compared to non-treated mdx mice (NT, *n* = 5). Data are presented as mean ± SD, ****P < 0.0001 compared to NT mice.

In vivo dystrophin expression was evaluated using fluorescence immunostaining using dystrophin antibodies. A high level of dystrophin expression was observed in TA muscle sections stained with dystrophin antibody 30 days post-IM injection of 2OMePS/PEI-loaded microspheres. The muscles were compared to normal levels of dystrophin expression found in WT mice (Fig. 5A). The amount of dystrophin was similar for both the WT and the IM microsphere-treated *mdx* mice. Controls of free 2OMePS polyplexes in *mdx* mice also resulted in dystrophin expression that was marginally lower than the microsphere-treated muscles. No dystrophin expression was observed in muscles injected with empty PF microspheres and non-treated *mdx* mice. The quantification of the dystrophin expression was performed using IMARIS image analysis software (Figure S4). For each image, dystrophin positive fibers were counted, and their fluorescence intensity was quantified and normalized to the fluorescence intensity background value obtained from the non-treated *mdx* mice specimens. Higher fluorescence intensity was observed after treatment with 2OMePS loaded PF microspheres (Fig. 5B). The number of dystrophin-positive fibers was counted and presented in Fig. 5C. An increase up to 92% of dystrophin-positive fibers was found after treatment with 2OMePS loaded PF microspheres, compared to 70% of dystrophin-positive fibers after injection with free 2OMePS polyplexes. This outcome is in agreement with the qPCR results showing higher exon skipping found in muscles injected with the 2OMePS loaded PF microspheres.

**Fig. 6:**
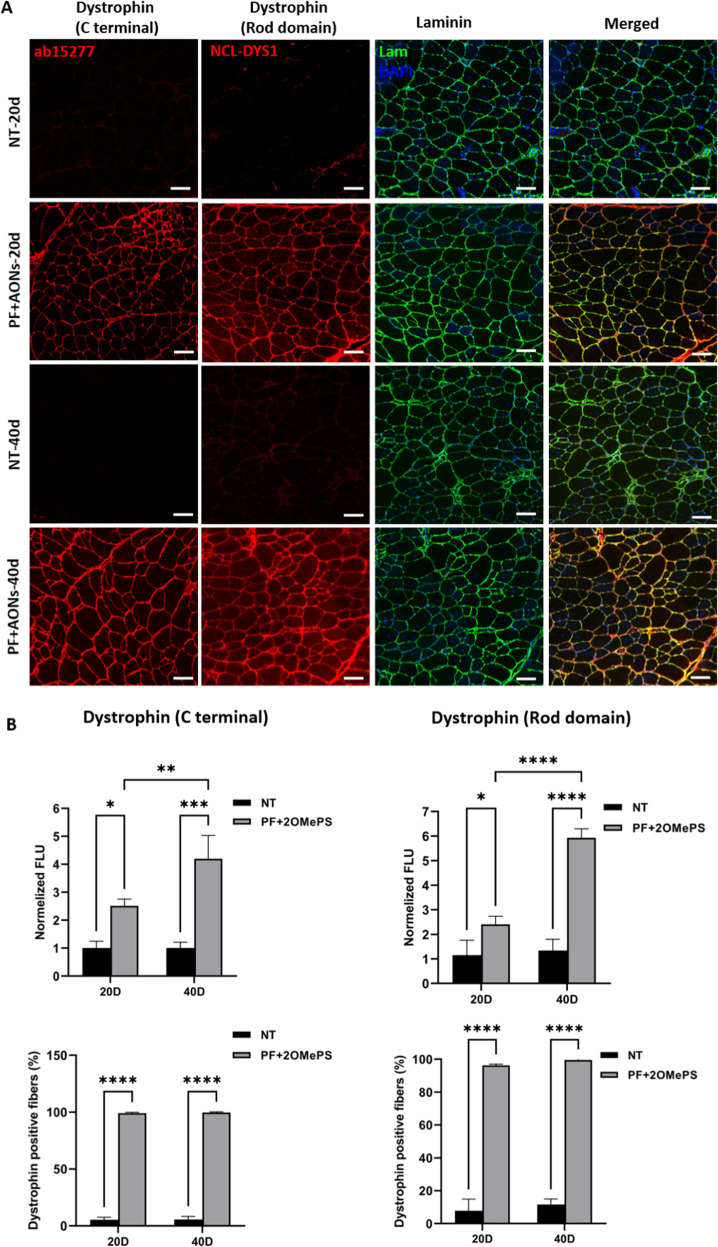
Dystrophin expression of TA muscles after intra-femoral (IF) injection. **A** Immunohistochemical staining of dystrophin (red) detected using ab15277 (C-term) and NCL- Dys1(Rod domain) 20 and 40 days post-Intra-femoral injection of 2OMePS/PEI loaded PF microspheres in *mdx* mice, compared to non-treated (NT) *mdx* mice at the same time points. Sections were counterstained for laminin to detect the sarcolemma (green), and DAPI to detect the nuclei (blue). Representative images are shown; scale bar = 50 μm. **B** Quantitative fluorescence intensity of 2OMePS/PEI loaded PF microsphere treatment normalized to non-treated (NT) mdx mice for the TA cryosections stained for dystrophin C-terminal region (ab15277) and ROD domain (NCL-DYS1). Quantitative data (*n* = 3) is presented as mean ± SD; ****P < 0.0001, ***P = 0.0001, **P = 0.0091, *P = 0.0169 compared to NT mdx mice. Also shown is the percentage of dystrophin-positive fibers in 2OMePS/PEI loaded PF microsphere treatment at 20 and 40 days post-Intra-femoral injection of *mdx* mice, compared to non-treated (NT) *mdx* mice. Quantitative data (*n* = 6) is presented as mean ± SD; ****P < 0.0001 compared to NT *mdx* mice.

TA cross-sections after IF injection of the 2OMePS loaded PF microspheres were analyzed for dystrophin expression using two types of antibodies: one binds to the C terminal region (ab15277) and the other to the rod domain region (NCL-DYS1) of the protein (Fig. 6A, Fig. S5). Staining of laminin antibody was done to visualize the fibers sarcolemma. Restoration of dystrophin 20 days and 40 days post-IF injection by 2OMePS-loaded PF microspheres was observed as indicated by sarcolemma localization at the C terminal and the rod domain regions of the protein. No dystrophin staining was observed in the non-treated *mdx* control mice. Quantitative analysis of fibers stained after 40 days post-IF injection with 2OMePS-loaded PF microspheres indicated significantly higher dystrophin expression when compared to non-treated controls (Fig. 6B). An increase of the fluorescence intensity by 2.5-fold and 4-fold was revealed in *mdx* mice treated by IF injection with 2OMePS-loaded PF microspheres, when compared to non-treated mice after 20 days and 40 days post-injection, respectively, using the C-terminal dystrophin antibody staining. There was nearly a 6-fold increase quantified in the slides stained with NCL-DYS1 antibody 40 days post-injection when comparing *mdx* mice treated by IF injection with 2OMePS-loaded PF microspheres to non-treated mice. Importantly, the percentage of dystrophin-positive fibers increased to nearly 100% after only 20 days post-injection, irrespective of the types of dystrophin antibody used. These findings indicate that the IF injection of 2OMePS with PF microspheres as a controlled released carrier enhances the efficacy of the AONs for higher dystrophin restoration.

Dystrophin expression after IF injection was evaluated by WB analysis (Fig. 4C). The *mdx* mice treated with an IF injection of 2OMePS loaded PF MPs were evaluated by WB after 20 days and compared to NT *mdx* controls. The WB results showed a similar dystrophin expression pattern to the immunohistochemical staining; namely that the treated muscles express more dystrophin protein compared to the NT mdx mice muscles. Importantly, the expression of dystrophin in the treated mdx mice muscles was still much less than the expression in WT muscles— an indication that full restoration was far from complete. This data was confirmed by Proteomic analysis using Mass Spectrometry (MS), which showed higher dystrophin levels in treated muscles compared to NT muscles in the mdx mice (data is not shown).

### Histological analysis of muscle sections

**Fig. 7:**
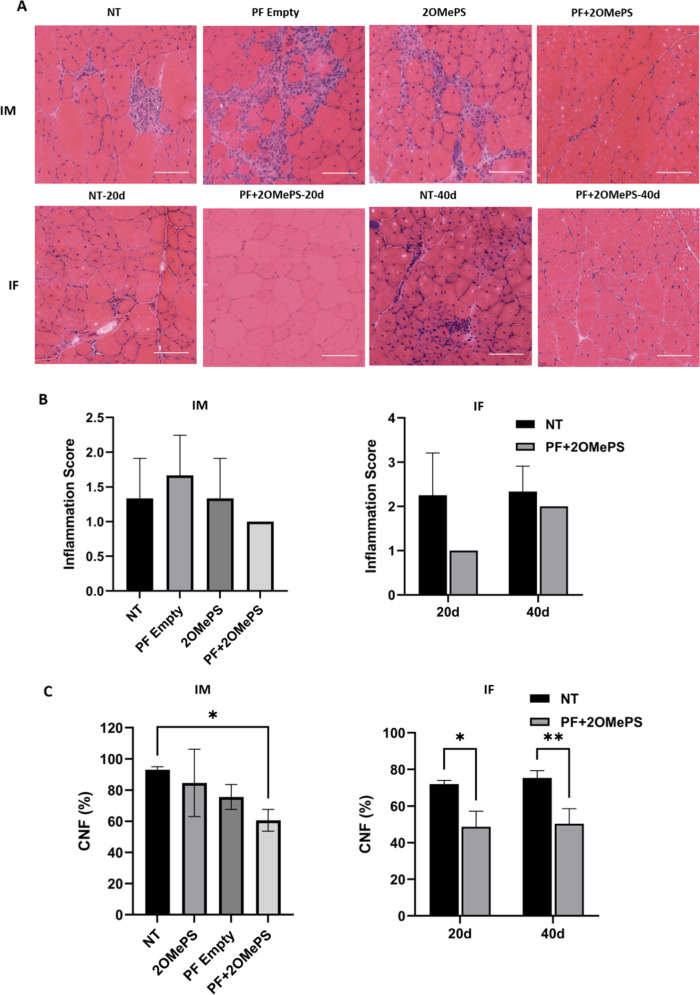
Histopathological analysis of muscles treated with 2OMePS/PEI polyplex-loaded PF microspheres. **A** H&E staining of cryosections of the TA muscles of mdx mice 30 days after intramuscular (IM) injection, empty PF microspheres (PF empty), free 2OMePs/PEI polyplexes (2OMePS) or 2OMePS/PEI loaded PF microspheres (PF + 2OMePS), and the non-treated (NT) group. Muscles treated by intra-femoral (IF) injection were also stained and compared to non-treated (NT) mdx mice 20- and 40-days post injections. The 20 days non-treated mdx mice (NT-20d) are compared to 20 days IF-treated mice (PF + 2OMePS-20d) and the 40 days non-treated mdx mice (NT-40d) are compared to 40 days IF treated mice (PF + 2OMePS-40d). Representative images are shown with a scale bar = 100 µm. **B** Average inflammation score analyzed from TA muscle sections after Intramuscular injection (IM) and intra-femoral injection (IF); the scoring date (*n* = 3) is presented as mean ± SD. **C** Percentage of centrally nucleated muscle fibers (CNFs) counted in TA cryosections after Intramuscular injection (IM) and intra-femoral injection (IF). Data (*n* = 3) is presented as mean ± SD; **P < 0.01, *P < 0.05 compared to NT mdx mice.

For the histological and pathological aspect of the muscles tissue at the time of sacrifice, the TA muscle sections were stained with H&E (Fig. 7A) and analyzed by an independent pathologist using a blinded scoring system (Fig. 7B). The muscles treated with 2OMePS/PEI-loaded PF microspheres were more homogenous in size when compared to the untreated *mdx* muscles (NT). The untreated *mdx* mice specimens display muscle pathologies including a variation of fiber size and immune cells infiltration foci (nuclei aggregates). Furthermore, muscle fibers with a small-caliber—an indication for muscle regeneration processes—were less prominent in the PF-loaded microsphere treated muscles after both IM and IF injections. The averaged minimal and maximal fiber sizes were larger in the PF-loaded microsphere treated muscles, compared to the non-treated muscles. This demonstrates a more mature and less regenerative fiber in the PF-treated muscles. This outcome was especially prominent in the IF injection (Fig. S6). The control group representing muscles treated with empty PF microspheres by IM injection showed mild to moderate inflammatory response as indicated by the H&E-stained images. The inflammatory phenotype increased the inflammation score of the empty PF treatment when compared to NT muscle; whereas a reduction in inflammation score was noted in muscles injected with the 2OMePS/PEI loaded microspheres. Less predictably, muscles injected with free 2OMePS/PEI polyplexes did not show any change in inflammation score compared to the non-treated group. When assessing the inflammation score after IF injection, muscles treated with the AON-laden PF microspheres showed the lowest inflammation score 20 days after the injection (Fig. 7B-left). This represents the mildest inflammation found in these tissues, whereas the NT mice exhibited a moderate inflammation at the same timepoint. Another unexpected outcome of the histopathology was observed at 40 days post-injection when there was only a slight improvement in the muscle’s inflammation compared to 20 days.

As is well established, dystrophic muscle fibers are prone to necrosis and undergoing recurring degeneration-regeneration processes that cause an increase in centrally nucleated muscle fibers (CNFs) [15, 62]. This dystrophic phenotype is also predominantly found in *mdx* mice muscles [63]. Thus, the number of CNFs in the TA section was quantified as a percentage of the total nuclei in the fibers (Fig. 7C). The number of CNFs was significantly reduced up to 60% in muscles of *mdx* mice injected IM with PF microspheres loaded with 2OMePS/PEI polyplexes. In IF injected muscles, there was a smaller percentage of CNFs; 54% after treatment with the 2OMePS/PEI polyplex loaded PF microspheres. These data indicate a decreased degeneration and regeneration and more mature fibers. Another important aspect to be considered is the apparent change in the fibrotic state of the tissue. This was visualized using Masson trichrome staining of the cryosections (Fig. S7). The presence of excess connective tissue found by collagen staining was a strong indication of fibrosis in the non-treated *mdx* mice muscle, and one of the important hallmarks of dystrophic muscle [64, 65]. In the IF injection muscles treated with PF-loaded microspheres after 20 and 40 days, there was a decrease in connective tissue areas as compared to non-treated *mdx* mice. This demonstrates a reduction in the fibrosis state of the muscle after treatment with 2OMePS/PEI polyplex-loaded PF microspheres.

There are limitations in using histological evaluations alone in order to assess muscle repair. The histological evaluations do not provide direct evidence of improved muscle function associated with dystrophin restoration. Further studies showing improvement in muscle function and performance after treatment should be explored to reinforce the potential of combining AON therapy with PF microspheres for treating DMD. This can be done using functionality and physical testing of treated *mdx* mice, including treadmill exercise tolerance testing or grip strength evaluation [66, 67]. These types of evaluations would require substantially longer follow-up periods to ensure dystrophin expression is restored and a relative abundance of protein is present in the treated muscles. The current study was limited to the early follow-up time-points where gene expression was confirmed but the protein levels in the muscle were limited. The restoration of the dystrophin expression after 20 and 40 days post-operatively coincided well with the persistence of the AON-bearing implanted MPs after 28 days, as indicated by MRI analysis. Longer term follow-up of the dystrophin expression in treated *mdx* mice should also be explored in future studies to verify the long-term implications of using MPs for AON therapy in DMD.

Beyond limitations of the current study, it is also important to note that exon skipping cannot comprehensively restore diseased muscle function in DMD patients. Using AONs aims to sequester a specific exon during splicing, resulting in a larger deletion and restoration of the reading frame. In DMD, the restored reading frame result is production of a slightly more functional dystrophin protein, rather than a nonfunctional one. The more functional dystrophin protein causes Becker’s Muscle Dystrophy (BMD), a more moderate form of the disease [68]. AON therapy is typically applied to mature muscle fibers to treat advanced stages of the disease, but recent evidence has shown that the absence of dystrophin in satellite cells (SC) can also alter asymmetric cell division, thus contributing to the progressive depletion of the stem cell pool in DMD individuals [69]. If AON therapy using 2OMePS loaded PF microspheres can also partially rescue division abnormalities in SCs, this could compound the beneficial effects by directly enhancing the SC populations in DMD patients. Further research is thus required to explore all the potential benefits of a controlled release approach in AON therapy for treating DMD.

## Conclusions

This study examined the potential of PF hydrogel microspheres to function as a sustained delivery vehicle for 2OMePS AONs when treating DMD. The results indicate that the administration of 2OMePS using PF microspheres in a controlled release manner enhanced the transfection efficiency of the encapsulated AONs, resulting in improved restoration of dystrophin after IM and IF administration in *mdx* mice muscles. A significantly higher exon skipping percentage in *mdx* mice injected by IF administration was demonstrated, suggesting that delivery of the AON-laden microspheres using IF injections can increase the overall efficacy of the AON therapy. Moreover, the treatment with PF microspheres improved the histopathological properties of the dystrophic muscles, indicating enhanced therapeutic benefits of this approach. The study, therefore, demonstrates that injectable PF microspheres for 2OMePS delivery can be applied either by IM or IF administration for DMD muscle restoration using an exon-skipping strategy.

## Supplementary information

Supplemental data

Author Checklist

## Data Availability

All datasets on which the conclusions of the paper rely will be made available to readers upon request.

## References

[1] Chung J, Smith AL, Hughes SC, Niizawa G, Abdel-Hamid HZ, Naylor EW (2016). Twenty-year follow-up of newborn screening for patients with muscular dystrophy. Muscle Nerve.

[2] Gao QQ, McNally EM (2015). The dystrophin complex: structure, function, and implications for therapy. Compr Physiol.

[3] Bushby K, Finkel R, Birnkrant DJ, Case LE, Clemens PR, Cripe L (2010). Diagnosis and management of Duchenne muscular dystrophy, part 2: implementation of multidisciplinary care. Lancet Neurol.

[4] Nelson CE, Hakim CH, Ousterout DG, Thakore PI, Moreb EA, Rivera RMC (2016). In vivo genome editing improves muscle function in a mouse model of Duchenne muscular dystrophy. Science (80-).

[5] Amoasii L, Hildyard JCW, Li H, Sanchez-Ortiz E, Mireault A, Caballero D. et al. Gene editing restores dystrophin expression in a canine model of duchenne muscular dystrophy. *Science (80-)* 2018. 10.1126/aau1549(2018). 10.1126/science.aau1549 PMC6205228 30166439

[6] Yuan J, Ma Y, Huang T, Chen Y, Peng Y, Li B (2018). Genetic modulation of RNA splicing with a CRISPR-guided cytidine deaminase. Mol Cell.

[7] Dzierlega K, Yokota T (2020). Optimization of antisense-mediated exon skipping for Duchenne muscular dystrophy. Gene Ther.

[8] Aoki Y, Yokota T, Nagata T, Nakamura A, Tanihata J, Saito T, et al. Bodywide skipping of exons 45-55 in dystrophic mdx52 mice by systemic antisense delivery. 10.1073/pnas.1204638109. 10.1073/pnas.1204638109 PMC3427064 22869723

[9] van Deutekom JC, Bremmer-Bout M, Janson AA, Ginjaar IB, Baas F, den Dunnen JT (2001). Antisense-induced exon skipping restores dystrophin expression in DMD patient derived muscle cells. Hum Mol Genet.

[10] Lu-Nguyen N, Dickson G, Malerba A. Systemic intravenous administration of antisense therapeutics for combinatorial dystrophin and myostatin exon splice modulation. In: *Methods in Molecular Biology*. 2018, pp 343–54. 10.1007/978-1-4939-8651-4_21 30171552

[11] Verheul RC, Van Deutekom JCT, Datson NA. Digital droplet PCR for the absolute quantification of exon skipping induced by antisense oligonucleotides in (Pre-)clinical development for duchenne muscular dystrophy. *PLoS ONE* 2016; **11**. 10.1371/journal.pone.0162467. 10.1371/journal.pone.0162467 PMC5017733 27612288

[12] Dias N, Stein CA (2002). Minireview antisense oligonucleotides: basic concepts and mechanisms. Mol Cancer Ther.

[13] Han G, Gu B, Cao L, Gao X, Wang Q, Seow Y, et al. ARTICLE Hexose enhances oligonucleotide delivery and exon skipping in dystrophin-deficient mdx mice. 2016. 10.1038/ncomms10981. 10.1038/ncomms10981 PMC4793046 26964641

[14] Han G, Lin C, Ning H, Gao X, Yin HF (2018). Long-term morpholino oligomers in hexose elicits long-lasting therapeutic improvements in mdx mice. Mol Ther - Nucleic Acids.

[15] Lu-Nguyen N, Malerba A, Popplewell L, Schnell F, Hanson G, Dickson G (2017). Systemic antisense therapeutics for dystrophin and myostatin exon splice modulation improve muscle pathology of adult mdx mice. Mol Ther - Nucleic Acids.

[16] Goemans NM, Tulinius M, van den Akker JT, Burm BE, Ekhart PF, Heuvelmans N (2011). Systemic administration of PRO051 in duchenne’s muscular dystrophy. N. Engl J Med.

[17] Shimizu-Motohashi Y, Miyatake S, Komaki H, Takeda S, Aoki Y (2016). Recent advances in innovative therapeutic approaches for Duchenne muscular dystrophy: From discovery to clinical trials. Am J Transl Res..

[18] Stein CA, Castanotto D (2017). FDA-approved oligonucleotide therapies in 2017. Mol Ther.

[19] Goemans N, Tulinius M, Kroksmark AK, Wilson R, van den Hauwe M, Campion G (2017). Comparison of ambulatory capacity and disease progression of Duchenne muscular dystrophy subjects enrolled in the drisapersen DMD114673 study with a matched natural history cohort of subjects on daily corticosteroids. Neuromuscul Disord.

[20] Evans CH, Huard J (2015). Gene therapy approaches to regenerating the musculoskeletal system. Nat Publ Gr.

[21] Carballo-Pedrares N, Fuentes-Boquete I, Díaz-Prado S, Rey-Rico A. Pharmaceutics Hydrogel-Based Localized Nonviral Gene Delivery in Regenerative Medicine Approaches-An Overview. 2020. 10.3390/pharmaceutics12080752. 10.3390/pharmaceutics12080752 PMC7464633 32785171

[22] Lei Y, Huang S, Sharif-Kashani P, Chen Y, Kavehpour P, Segura T (2010). Incorporation of active DNA/cationic polymer polyplexes into hydrogel scaffolds. Biomaterials.

[23] Lei Y, Rahim M, Ng Q, Segura T (2011). Hyaluronic acid and fibrin hydrogels with concentrated DNA/PEI polyplexes for local gene delivery. J Control Release.

[24] Yun YH, Goetz DJ, Yellen P, Chen W (2004). Hyaluronan microspheres for sustained gene delivery and site-specific targeting. Biomaterials.

[25] Tokatlian T, Cam C, Siegman SN, Lei Y, Segura T (2012). Design and characterization of microporous hyaluronic acid hydrogels for in vitro gene transfer to mMSCs. Acta Biomater.

[26] Kong HJ, Kim ES, Huang Y-C, Mooney DJ. Design of biodegradable hydrogel for the local and sustained delivery of angiogenic plasmid DNA. 10.1007/s11095-007-9526-7. 10.1007/s11095-007-9526-7 18183476

[27] Alam P, Haile B, Arif M, Pandey R, Rokvic M, Nieman M, et al. Inhibition of senescence-associated genes Rb1 and Meis2 in adult cardiomyocytes results in cell cycle reentry and cardiac repair post–myocardial infarction. *J Am Heart Assoc.* 2019; **8**. 10.1161/JAHA.119.012089. 10.1161/JAHA.119.012089 PMC6761626 31315484

[28] Needham CJ, Shah SR, Dahlin RL, Kinard LA, Lam J, Watson BM (2014). Osteochondral tissue regeneration through polymeric delivery of DNA encoding for the SOX trio and RUNX2. Acta Biomater.

[29] Li Y, Yang C, Khan M, Liu S, Hedrick JL, Yang YY (2012). Nanostructured PEG-based hydrogels with tunable physical properties for gene delivery to human mesenchymal stem cells. Biomaterials.

[30] Chun KW, Lee JB, Kim SH, Park TG (2005). Controlled release of plasmid DNA from photo-cross-linked pluronic hydrogels. Biomaterials.

[31] Kabanov A, Zhu J, Alakhov V (2005). Pluronic block copolymers for gene delivery. Adv Genet.

[32] Cohen SA, Simaan-Yameen H, Fuoco C, Gargioli C, Seliktar D (2022). Injectable hydrogel microspheres for sustained gene delivery of antisense oligonucleotides to restore the expression of dystrophin protein in duchenne muscular dystrophy. Eur Polym J.

[33] Ehrhardt C, Schmolke M, Matzke A, Knoblauch A, Will C, Wixler V (2006). Polyethylenimine, a cost-effective transfection reagent. Signal Transduct.

[34] Ko YT, Bickel U, Huang J (2011). Polyethylenimine/oligonucleotide polyplexes investigated by fluorescence resonance energy transfer and fluorescence anisotropy. Oligonucleotides.

[35] Lungwitz U, Breunig M, Blunk T, Göpferich A (2005). Polyethylenimine-based non-viral gene delivery systems. Eur J Pharm Biopharm.

[36] Pradhan S, Clary JM, Seliktar D, Lipke EA (2017). A three-dimensional spheroidal cancer model based on PEG-fibrinogen hydrogel microspheres. Biomaterials.

[37] Franco CL, Price J, West JL (2011). Development and optimization of a dual-photoinitiator, emulsion-based technique for rapid generation of cell-laden hydrogel microspheres. Acta Biomater.

[38] Gonin P, Arandel L, Van Wittenberghe L, Marais T, Perez N, Danos O (2005). Femoral intra-arterial injection: a tool to deliver and assess recombinant AAV constructs in rodents whole hind limb. J Gene Med.

[39] Greelish JP, Su LT, Lankford EB, Burkman JM, Chen H, Konig SK (1999). Stable restoration of the sarcoglycan complex in dystrophic muscle perfused with histamine and a recombinant adeno-associated viral vector. Nat Med.

[40] Fletcher S, Honeyman K, Fall AM, Harding PL, Johnsen RD, Wilton SD (2006). Dystrophin expression in themdx mouse after localised and systemic administration of a morpholino antisense oligonucleotide. J Gene Med.

[41] Lu QL, Rabinowitz A, Chen YC, Yokota T, Yin H, Alter J (2005). Systemic delivery of antisense oligoribonucleotide restores dystrophin expression in body-wide skeletal muscles. Proc Natl Acad Sci USA.

[42] Lu QL, Bou-Gharios G, Partridge TA (2003). Non-viral gene delivery in skeletal muscle: a protein factory. Gene Ther.

[43] Sampaolesi M, Torrente Y, Innocenzi A, Tonlorenzi R, D’Antona G, Pellegrino MA (2003). Cell therapy of α-sarcoglycan null dystrophic mice through intra-arterial delivery of mesoangioblasts. Science (80-).

[44] Gargioli C, Coletta M, De Grandis F, Cannata SM, Cossu G (2008). PlGF-MMP-9-expressing cells restore microcirculation and efficacy of cell therapy in aged dystrophic muscle. Nat Med.

[45] Boussif O, Lezoualc’h F, Zanta MA, Mergny MD, Scherman D, Demeneix B (1995). A versatile vector for gene and oligonucleotide transfer into cells in culture and in vivo: polyethylenimine. Proc Natl Acad Sci USA.

[46] Dikovsky D, Bianco-Peled H, Seliktar D (2006). The effect of structural alterations of PEG-fibrinogen hydrogel scaffolds on 3-D cellular morphology and cellular migration. Biomaterials.

[47] Boldrin L, Zammit PS, Morgan JE (2015). Satellite cells from dystrophic muscle retain regenerative capacity. Stem Cell Res.

[48] Sarig R, Baruchi Z, Fuchs O, Nudel U, Yaffe D (2006). Regeneration and transdifferentiation potential of muscle-derived stem cells propagated as myospheres. Stem Cells.

[49] Berdichevski A, Yameen HS, Dafni H, Neeman M, Seliktar D (2015). Using bimodal MRI/fluorescence imaging to identify host angiogenic response to implants. Proc Natl Acad Sci USA.

[50] Pradhan S, Hassani I, Seeto WJ, Lipke EA (2017). PEG-fibrinogen hydrogels for three-dimensional breast cancer cell culture. J Biomed Mater Res - Part A.

[51] Akinc A, Thomas M, Klibanov AM, Langer R (2005). Exploring polyethylenimine-mediated DNA transfection and the proton sponge hypothesis. J Gene Med.

[52] Lei Y, Segura T (2009). DNA delivery from matrix metalloproteinase degradable poly(ethylene glycol) hydrogels to mouse cloned mesenchymal stem cells. Biomaterials.

[53] Frisman I, Seliktar D, Bianco-Peled H. Nanostructuring PEG-fibrinogen hydrogels to control cellular morphogenesis. *Biomaterials* 2011. 10.1016/j.biomaterials.2011.06.078. 10.1016/j.biomaterials.2011.06.078 21784517

[54] Partridge TA (2013). The mdx mouse model as a surrogate for Duchenne muscular dystrophy. FEBS J.

[55] Telgmann L, Sperling M, Karst U (2013). Determination of gadolinium-based MRI contrast agents in biological and environmental samples: a review. Anal Chim Acta.

[56] Goyenvalle A, Griffith G, Babbs A, Andaloussi SEL, Ezzat K, Avril A (2015). Functional correction in mouse models of muscular dystrophy using exon-skipping tricyclo-DNA oligomers. Nat Med.

[57] Novak JS, Hogarth MW, Boehler JF, Nearing M, Vila MC, Heredia R, et al. Myoblasts and macrophages are required for therapeutic morpholino antisense oligonucleotide delivery to dystrophic muscle. 10.1038/s41467-017-00924-7. 10.1038/s41467-017-00924-7 PMC5643396 29038471

[58] Scaglioni D, Catapano F, Ellis M, Torelli S, Chambers D, Feng L (2021). The administration of antisense oligonucleotide golodirsen reduces pathological regeneration in patients with Duchenne muscular dystrophy. Acta Neuropathol Commun.

[59] Kim D-H, Chen J, Omary RA, Larson AC (2015). MRI visible drug eluting magnetic microspheres for transcatheter intra-arterial delivery to liver tumors. Theranostics.

[60] Hong K, Khwaja A, Liapi E, Torbenson MS, Georgiades CS, Geschwind J-FH (2006). New intra-arterial drug delivery system for the treatment of liver cancer: preclinical assessment in a rabbit model of liver cancer. Clin Cancer Res.

[61] Gallo JM, Gupta PK, Hung CT, Perrier DG (1989). Evaluation of drug delivery following the administration of magnetic albumin microspheres containing adriamycin to the rat. J Pharm Sci.

[62] Van Putten M, Hulsker M, Nadarajah VD, Van Heiningen SH, Van, Huizen E (2012). The effects of low levels of dystrophin on mouse muscle function and pathology. PLoS ONE.

[63] McGreevy JW, Hakim CH, McIntosh MA, Duan D (2015). Animal models of Duchenne muscular dystrophy: from basic mechanisms to gene therapy. DMM Dis Model Mech..

[64] Rojas-Marcos I. Muscular dystrophies. *Med* 2019; **12**. 10.1016/j.med.2019.04.003.

[65] Ogura Y, Tajrishi MM, Sato S, Hindi SM, Kumar A (2014). Therapeutic potential of matrix metalloproteinases in Duchenne muscular dystrophy. Front cell Dev Biol.

[66] Malerba A, Sharp PS, Graham IR, Arechavala-Gomeza V, Foster K, Muntoni F (2011). Chronic systemic therapy with low-dose morpholino oligomers ameliorates the pathology and normalizes locomotor behavior in mdx mice. Mol Ther.

[67] Denti MA, Rosa A, D’Antona G, Sthandier O, De Angelis FG, Nicoletti C (2006). Body-wide gene therapy of Duchenne muscular dystrophy in the mdx mouse model. Proc Natl Acad Sci USA.

[68] Niks EH, Aartsma-Rus A. Exon skipping: a first in class strategy for Duchenne muscular dystrophy. 101080/1471259820171271872 2016; **17**: 225–36. 10.1080/14712598.2017.1271872 27936976

[69] Dumont NA, Wang YX, Von Maltzahn J, Pasut A, Bentzinger CF, Brun CE (2015). Dystrophin expression in muscle stem cells regulates their polarity and asymmetric division. Nat Med.

