# Supplementary material for: In vivo restoration of dystrophin expression in mdx mice using intra-muscular and intra-arterial injections of hydrogel microsphere carriers of exon skipping antisense oligonucleotides
Source: Cell Death Dis. 2022 Sep 9;13(9):779. doi: 10.1038/s41419-022-05166-0 (PMC9463190; doi:10.1038/s41419-022-05166-0)
Supplement: Supplementary file 1 — Supplemental data [file 41419_2022_5166_MOESM1_ESM.docx]

**Supplementary Figures**


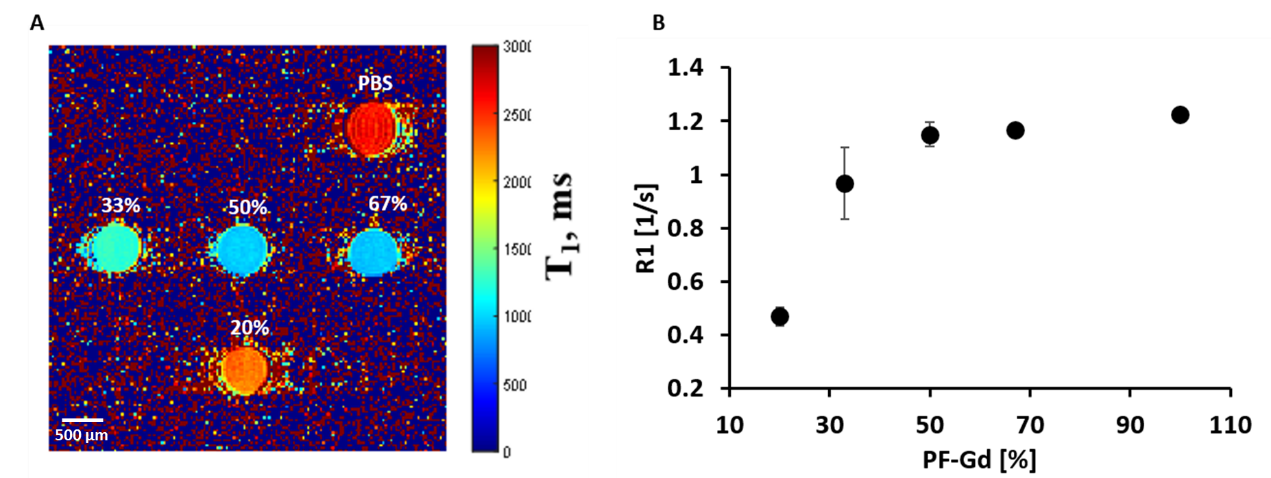


**Figure S1:** **(A)** T1 color map of the PF-GdDTPA in PBS solution and **(B)** R1 relaxivity values of PF-GdDTPA as a function PF-Gd percentage in PBS solution. An increase in the R1 relaxation constant was observed with increasing concentration of Gd labeled PF microspheres in PBS solution.


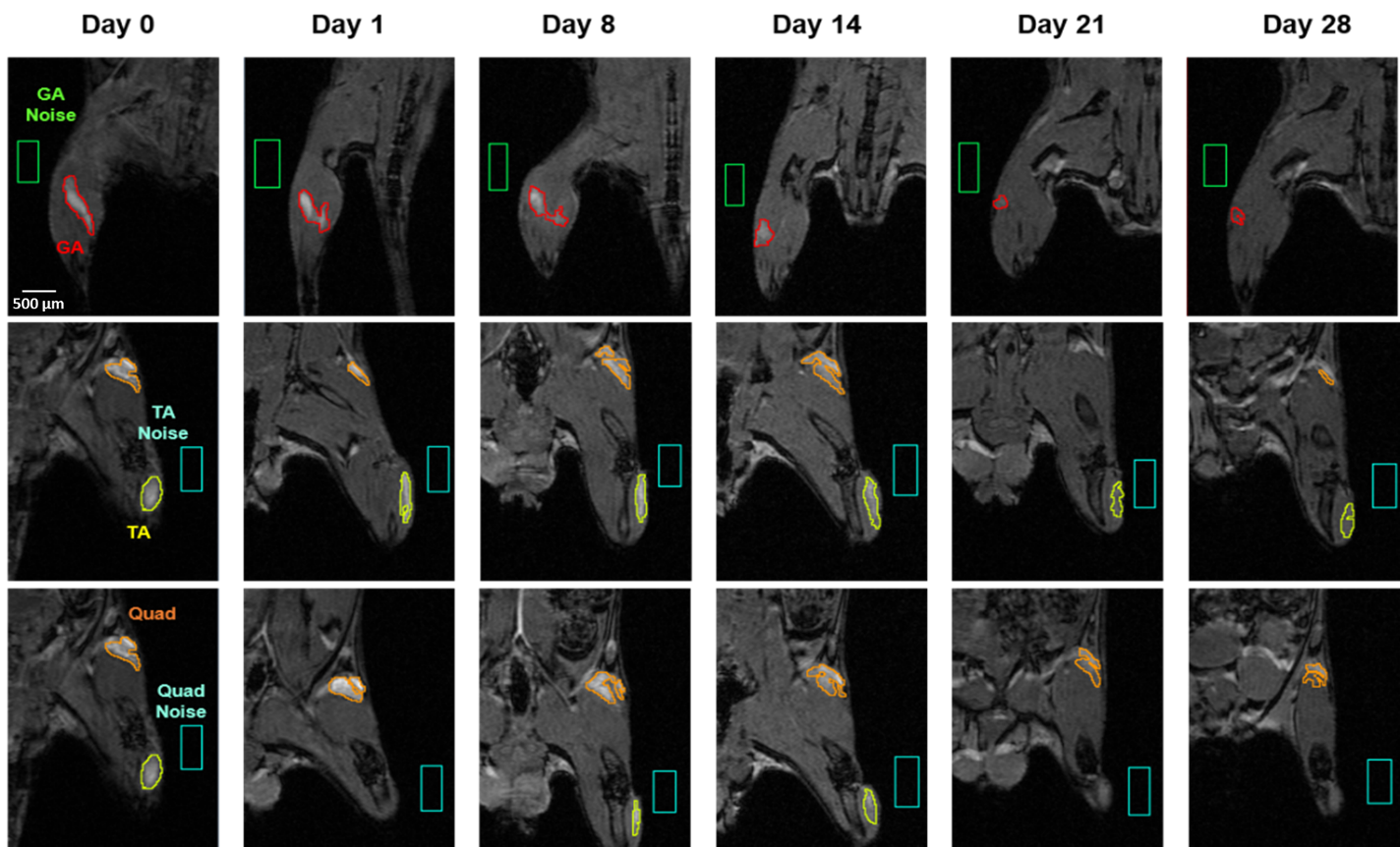


**Figure S2:** Representative MRI images of mdx mice injected with Gd labeled PF microspheres. The GA (up), TA (middle), and quadriceps (down) were injected with 30 ul of PF microspheres..The mdx mice were imaged using MRI several time points post-injection and the signal to noise ratio (SNR) in each time point was calculated using MIPAV program. Reduction of the labeled PF microspheres signal was observed in all three muscles demonstrating the in vivo biodegradation of the microspheres hydrogel up to 28 days post-injection.


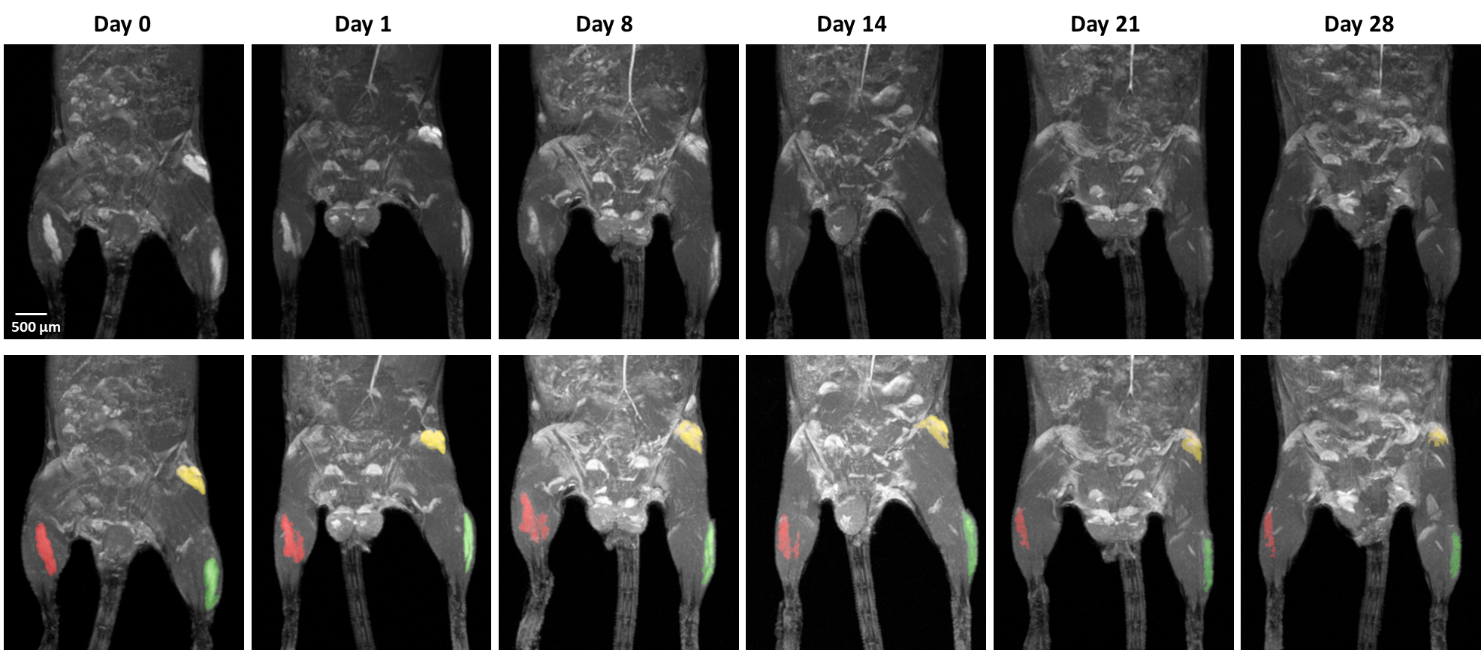


**Figure S3:** 3D MRI reconstructions of C57-WT mice injected with Gd labeled PF microspheres in the GA, TA and Quadriceps muscles. At the bottom false colors used to identify implants for each muscle group - GA in red, TA in green, and Quadriceps in yellow showing the volume reduction of the MPs over time used for registration.


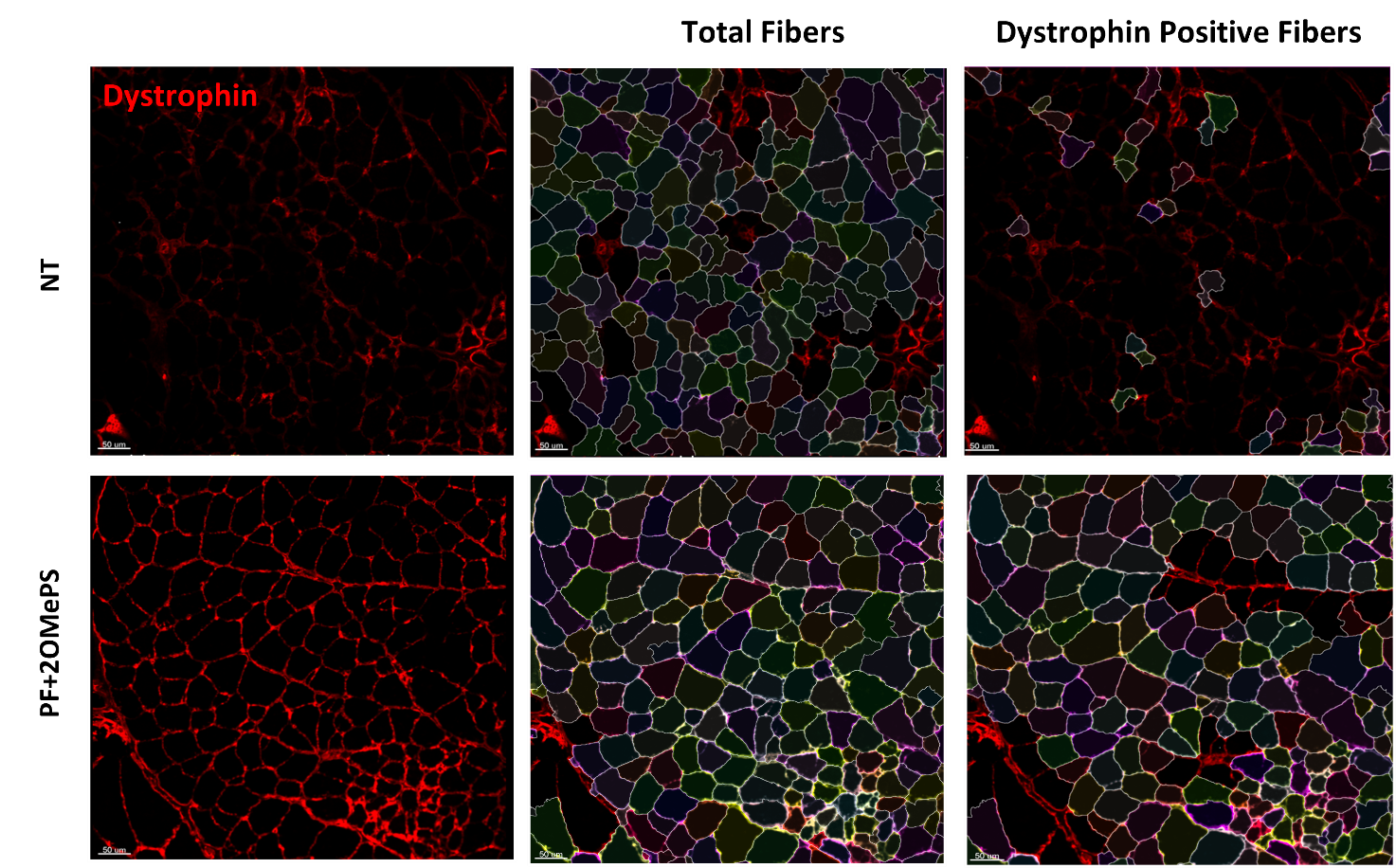


**Figure S4:** Dystrophin positive fibers assessment using IMARIS image analysis. Represented images of dystrophin-stained TA muscles sections of non-treated mdx mice (up) and mdx mice treated with PF microspheres loaded with 2OMePS/PEI polyplexes (down) 30 days post intramuscular injection. Scale bar=50 µm.


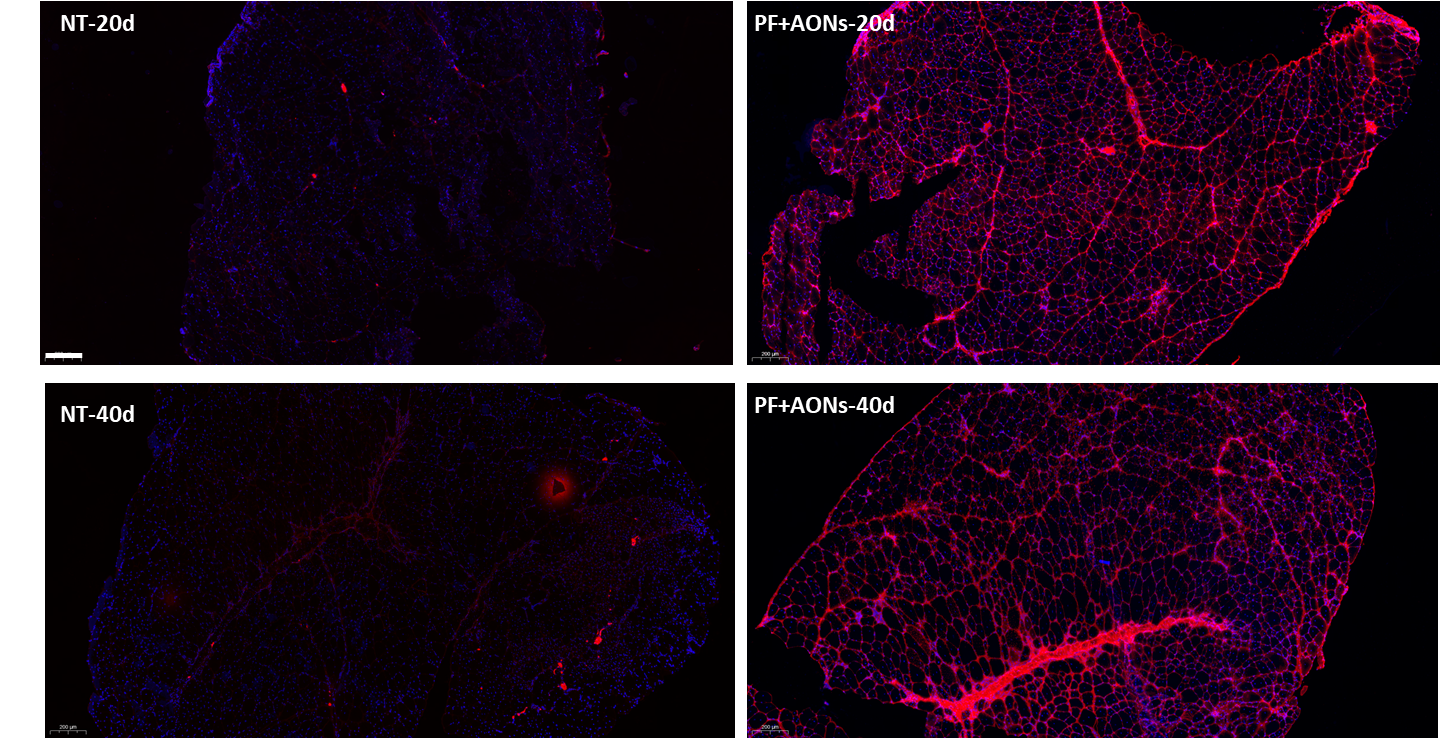


**Figure S5**: X5 magnification of immunohistochemical staining of dystrophin (red) detected using ab15277 20 and 40 days post-Intra-femoral injection of 2OMePS/PEI loaded PF microspheres in mdx mice, compared to non-treated (NT) mdx mice at the same time points, scale bar=200 μm.

**Histology Analysis**:

A semi-quantitative analysis was performed, to evaluate the cellular inflammation and the fibrosis of the tissues according to the following criteria:

•**Cellular inflammation** (number of cells, per X20 mag.):

Grade 0: Normal.

Grade 1: Mild -10- 20 cells

Grade 2: Moderate - 20-50 cells

Grade 3: Severe - More than 50 cells

•**Fibrosis** (per x20 magnification field in affected areas):

Grade 0: Normal

Grade 1: Mild, up to 25% of the tissue sample

Grade 2: Moderate, 25-75% of the tissue sample

Grade 3: Severe, more than 75%


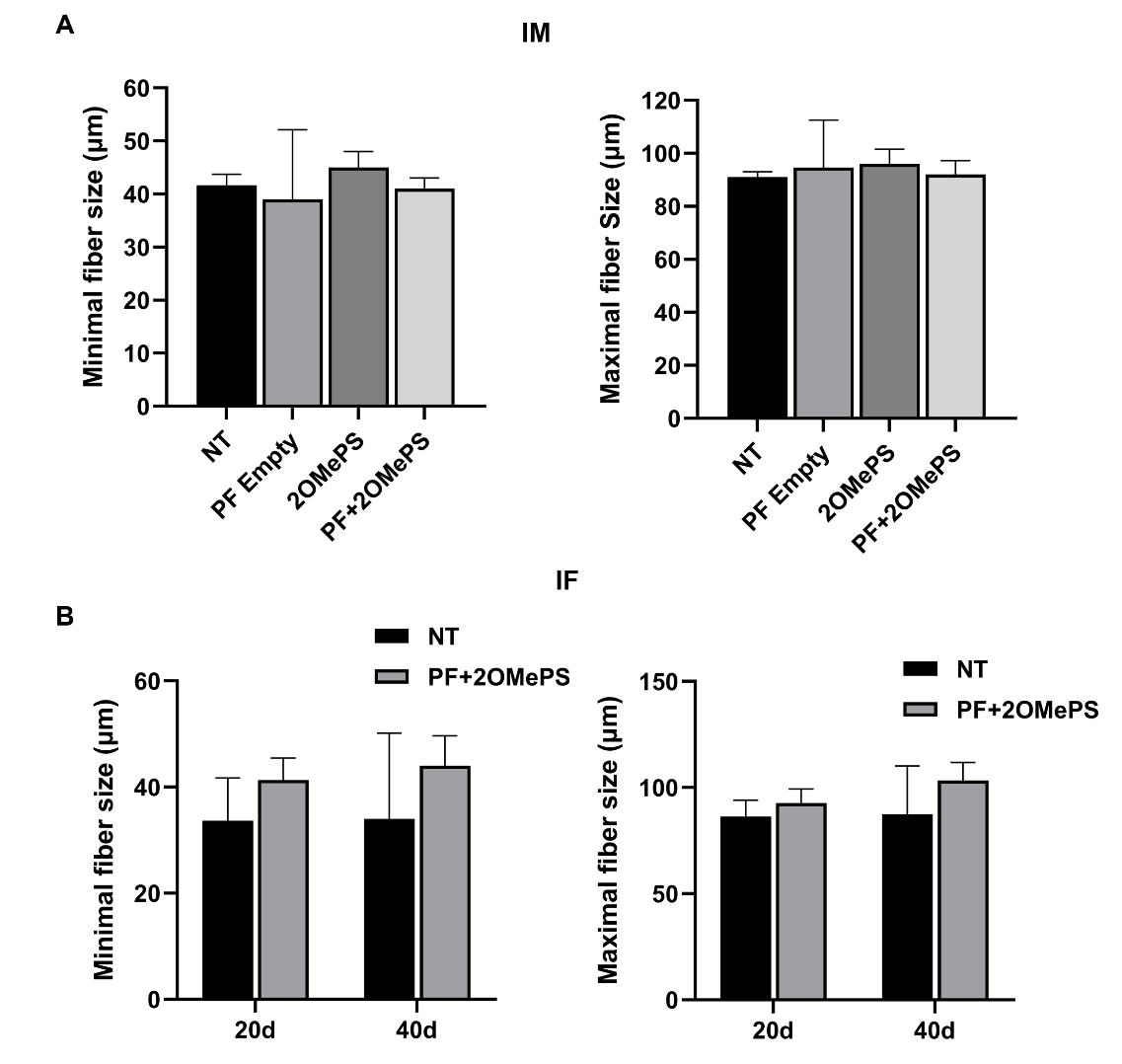


**Figure S6:** Average minimal and maximal fibers diameter of H&E-stained TA cryosections 30 days post intramuscular (IM) injection **(A)** or 20 and 40 days post-intra-femoral (IF) injection. The average diameter was measured in micrometers from H&E staining images of the muscles and the mean of 10 measurements per cross section was calculated. The minimal and maximal size of muscles treated with 2OMePS loaded PF microspheres showed a slight increase in size. However, relative bigger fibers were measured after IF injection observed 20 days and 40 days post injection compared to the non-treated mice, sacrificed at the same day. These outcomes demonstrate that treatment with PF loaded microspheres results with more mature and less regenerative fibers with improved pathological parameter found after IF injection.


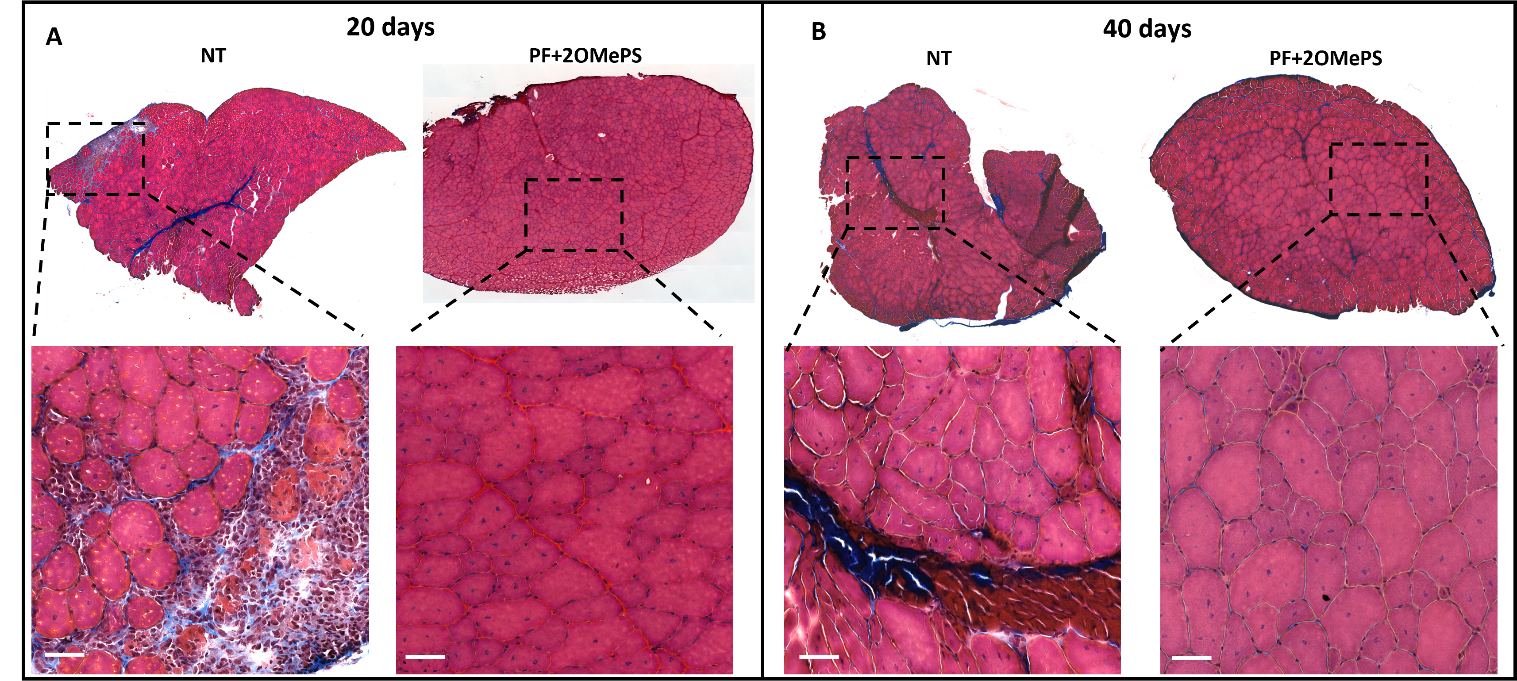


**Figure S7:** Representative images of Masson trichrome staining of TA muscles after intra-femoral injection of 2OMePS/PEI loaded microspheres 20 **(A)** and 40 days **(B)** post-injection. The fibrosis state of the muscles tissue was examined showing the decreased presence of stained connective tissue and fat as demonstrated after treatment with PF microspheres 20 days and 40 days post-injection compared to the non-treated mice. Scale bar=50 µm.
